# Factors influencing the adoption of an innovation: An examination of the uptake of the Canadian Heart Health Kit (HHK)

**DOI:** 10.1186/1748-5908-3-41

**Published:** 2008-10-02

**Authors:** Shannon D Scott, Ronald C Plotnikoff, Nandini Karunamuni, Raphaël Bize, Wendy Rodgers

**Affiliations:** 1 Faculty of Nursing, University of Alberta, Edmonton, AB, Canada; 2 Centre for Health Promotion Studies, School of Public Health, University of Alberta, Edmonton, AB, Canada; 3 Faculty of Physical Education & Recreation, University of Alberta, Edmonton, AB, Canada; 4 Alberta Centre for Active Living, University of Alberta, Edmonton, AB, Canada; 5 Department of Ambulatory Care and Community Medicine, University of Lausanne, Lausanne, Switzerland

## Abstract

**Background:**

There is an emerging knowledge base on the effectiveness of strategies to close the knowledge-practice gap. However, less is known about how attributes of an innovation and other contextual and situational factors facilitate and impede an innovation's adoption. The Healthy Heart Kit (HHK) is a risk management and patient education resource for the prevention of cardiovascular disease (CVD) and promotion of cardiovascular health. Although previous studies have demonstrated the HHK's content validity and practical utility, no published study has examined physicians' uptake of the HHK and factors that shape its adoption.

**Objectives:**

Conceptually informed by Rogers' Diffusion of Innovation theory, and Theory of Planned Behaviour, this study had two objectives: (1) to determine if specific attributes of the HHK as well as contextual and situational factors are associated with physicians' intention and actual usage of the HHK kit; and (2), to determine if any contextual and situational factors are associated with individual or environmental barriers that prevent the uptake of the HHK among those physicians who do not plan to use the kit.

**Methods:**

A sample of 153 physicians who responded to an invitation letter sent to all family physicians in the province of Alberta, Canada were recruited for the study. Participating physicians were sent a HHK, and two months later a study questionnaire assessed primary factors on the physicians' clinical practice, attributes of the HHK (relative advantage, compatibility, complexity, trialability, observability), confidence and control using the HHK, barriers to use, and individual attributes. All measures were used in path analysis, employing a causal model based on Rogers' Diffusion of Innovations Theory and Theory of Planned Behaviour.

**Results:**

115 physicians (follow up rate of 75%) completed the questionnaire. Use of the HHK was associated with intention to use the HHK, relative advantage, and years of experience. Relative advantage and the observability of the HHK benefits were also significantly associated with physicians' intention to use the HHK. Physicians working in solo medical practices reported experiencing more individual and environmental barriers to using the HHK.

**Conclusion:**

The results of this study suggest that future information innovations must demonstrate an advantage over current resources and the research evidence supporting the innovation must be clearly visible. Findings also suggest that the innovation adoption process has a social element, and collegial interactions and discussions may facilitate that process. These results could be valuable for knowledge translation researchers and health promotion developers in future innovation adoption planning.

## Background

'Knowledge translation,' the scientific study of the methods for closing the knowledge-to-practice gap, has emerged as a potential answer to the challenge of improving the quality of health care and patient outcomes [1]. In recent years, the terms 'knowledge translation,' 'research implementation,' 'evidence-based medicine,' and 'evidence-based decision making' have become conventional monikers in the health system [2-4]. Understanding factors that could influence the adoption of new ideas and innovations is an important step in efficient dissemination of potential innovations. Furthermore, social-cognitive theories could be utilized in understanding and implementing behaviour change/behaviour adoption interventions.

Factors that influence the adoption of the Healthy Heart Kit (HHK) by physicians can be explored using a theoretical premise. HHK was developed in 1999 by the Adult Health Division of Health Canada to ensure physicians have the latest knowledge for the prevention of CVD and promotion of cardiovascular health. This HHK is a risk management and patient education resource as well as a manual prevention reminder system. The kit was endorsed by the "Achieving Cardiovascular Health in Canada" intersectoral collaboration through meeting the Canadian Medical Association standard of guidelines for cardiovascular health promotion. The HHK includes appropriate patient education brochures and chart stickers as paper-based reminders. The kit targets the following CVD risk factors: smoking, obesity/overweight, sedentary lifestyle, hypercholesterolemia, hypertension, and diabetes mellitus.

*Rogers' Diffusion of Innovation Theory *[5] seeks to explain how new ideas or innovations (such as the HHK) are adopted, and this theory proposes that there are five attributes of an innovation that effect adoption: (1) relative advantage, (2) compatibility, (3) complexity, (4) trialability, and (5), observability. *Relative advantage *is the degree to which an innovation is perceived as being better than the idea it supersedes. Rogers' theory suggests that innovations that have a clear, unambiguous advantage over the previous approach will be more easily adopted and implemented. Current research evidence indicates that if a potential user sees no relative advantage in using the innovation, it will not be adopted [6]. *Compatibility *is the degree to which an innovation fits with the existing values, past experiences, and needs of potential adopters. There is strong direct research evidence suggesting that the more compatible the innovation is, the greater the likelihood of adoption [6]. *Complexity *is the degree to which an innovation is perceived as difficult to understand and use. Furthermore, Rogers suggested that new innovations may be categorized on a complexity-simplicity continuum with a qualification that the meaning (and therefore the relevance) of the innovation may not be clearly understood by potential adopters. When key players perceive innovations as being simple to use the innovations will be more easily adopted [6]. *Trialability *is the degree to which an innovation may be experimented with on a limited basis. Because new innovations require investing time, energy and resources, innovations that can be tried before being fully implemented are more readily adopted. And finally, *observability *is the degree to which the results of an innovation are visible to the adopters. If there are observable positive outcomes from the implementation of the innovation then the innovation is more adoptable.

Several social psychological theories suggest that the most immediate and important predictor of a person's behaviour (such as adoption of the HHK) is his/her intention to perform it (such as intending to use the HHK). Theory of Planned Behaviour (TPB) proposes that a person's intention to perform behaviour is the central determinant of that behaviour because it reflects the level of motivation a person is willing to exert to perform the behaviour [7]. The TPB has been largely used by researchers to understand a variety of health-related behaviours in various population groups. Eccles and colleagues [8] suggest that there is a predictable link between health care professionals' intention to engage in behaviour and their subsequent behaviour.

Conceptually informed by Rogers' Diffusion of Innovation theory, and the intention-behaviour association (based on the TPB), this study had two objectives: (1) to determine if specific attributes of the HHK as well as contextual and situational factors are associated with physicians' intention and actual usage of the kit; (2) if any contextual and situational factors are associated with individual or environmental barriers that prevent the uptake of the HHK among those physicians who do not intend to use the kit.

## Methods

### Sample

Physicians who responded (n = 153) to an invitation letter sent to all family physicians registered with the College of Physicians and Surgeons (n = 3068, at the time of the study) within the province of Alberta, Canada were recruited for the study. Inclusion criterion for the study was having at least a 0.5 FTE (full-time equivalent) position. Physicians who had been previously exposed to the HHK were excluded. Participating physicians were sent a HHK and then two months after a study questionnaire was mailed along with a stamped, self-addressed envelope. A more detailed account of the study methods are presented elsewhere [9].

### Questionnaire

The theoretical underpinnings of the questionnaire were based on Rogers' Diffusion of Innovation theory and the TPB. The questionnaire assessed nine primary factors: 1) physicians' clinical behaviours in terms of current cardiovascular health promotion (i.e., calculating coronary heart disease risk, promotion of healthy eating), 2) physicians' knowledge of the Alberta Medical Association guideline for "Management of Modifiable Risk Factors in Adults at High Risk for Cardiovascular Events" and the HHK, 3) the number of clinical hours per week and average length of patient encounter, 4) the attributes of the HHK and the above mentioned guideline, 5) physicians' confidence and control using the HHK, 6) physicians' likelihood of clinical practice change and use of HHK, 7) physicians' perspectives on barriers to using the HHK, 8) information about the physicians' clinical practice, and 9) individual physician attributes (e.g., educational background, smoking history, exercise behaviour). Individual items used to measure the above factors are described below.

Physicians' clinical behaviours in terms of cardiovascular health promotion was assessed by asking the doctors to describe the frequency with which they deliver the following services to their patients: weigh patients; calculate their BMI; calculate their Coronary Heart Disease Risk; counsel patients to cease smoking; counsel patients to increase physical activity; counsel patients to improve their diets. These were measured ordinally using a four-point scale from 'never' to 'frequently.' 'Physicians' knowledge of the guideline and HHK was assessed by asking "Before your enrolment in this study, were you aware of the 'Guidelines for Management of Modifiable Risk Factors in Adults at High Risk for Cardiovascular Events' published by the Alberta Medical Association," and "were you aware of the existence of the HHK." Both of these questions had the response options of 'yes' or 'no' and if yes, they were asked "how did you first learn about these guidelines or the HHK?" The number of clinical hours per week and average length of patient encounter duration was assessed with two items: "number of hours per week spent in patient care" with the following response options: < 20; 20–40; > 40; and "on average, what is the duration of your encounters with your patients?" with the following response options: 0–5 mins; 6–10 min; 11–15 min; 16–20 min; 21–25 min; 26–30 min; > 30 min. This item was dichotomized to short or long duration (less than 15 mins = 1; more than 15 mins = 2).

Attributes of the innovations was assessed using the following five constructs: *Relative advantage *was measured using the item "using the kit is more effective than our current practice" with the response options: strongly agree (5) to strongly disagree (1). *Compatibility *was assessed using the following three items with the same response options as above. The items were "the content of the kit is compatible with my personal beliefs and values"; "the kit is useful" and "the kit is credible." *Complexity *was assessed using the items: "the kit is easy/simple to use;" "the content of the kit is clear;" "the content of the kit is relevant" with the same response options as above. *Trialability *was measured using the items "the kit can be experimented without requiring an extensive involvement" and "the kit can be adapted or modified to suit my own needs" with the same response options. *Observability *was assessed with the items "the benefits of using the kit with my patients are obvious/visible" and "the evidence regarding the impact of using the kit on practice is available" with the same response options. The item "the evidence regarding the impact of using the kit on practice is available" was conceptualized as part of observability as we understood research on the effects of using the HHK to be a component of being able to observe or see the effects of the kit. The item "how much would using the 'HHK' be under your control," was measured on a nine-point scale with response options ranging from "having very little control" (1) to "having complete control" (9), and the item "how confident are you that you could use the HHK" was measured with response options ranging from "being not at all confident" (1) to "being completely confident," (9).

*Behavioural intention *[7] was measured with the item "How likely is it that you will change your practice as a result of the HHK" and *behaviour *was assessed by asking the participants "How often did you use the HHK with appropriate patients since you received it from us." For the above two questions, participants were asked to mark an X on a horizontal scale ranging from 0% (almost never) to 100% (almost certain).

Physicians' perspectives on the barriers to using the HHK were assessed using the item, "if you do not intend to use the 'HHK' on a regular basis in your practice, what are your reasons for not doing so. This was measured ordinally on a 5 point scale with the following categories which have been categorized as either individual or environmental barriers: no advantage to current practice (individual); not a priority area for me (individual); insufficient time to implement (individual and environmental); policies in my organizations prevent changes(environmental); require more resources for implementation (environmental); not feasible in my normal daily work (individual); not relevant for my patients (individual); lack of consensus amongst my colleagues (environmental); lack of knowledge in this particular area (individual). Setting of the practice (*Solo vs Group*) was assessed by asking the participants to choose from the following response options: Outpatient/walk-in clinic; Solo-practice; Group practice; Clinic associated with a tertiary/acute care setting or other (specify). This item was dichotomized to obtain solo vs. group practice (1 = solo; 2 = group). And finally individual physician attributes, such as educational background, years of experience, smoking history and exercise behaviour, were also assessed. *Years of experience *was assessed by asking the participants to choose the "year of graduation from University (Medical Degree)" in 10 year intervals.

The study received university-based ethics review clearance, as well as institutional permission from the Alberta Medical Association to access a list of their family physicians in order to recruit participants for the study.

### Analysis

Statistical Package for the Social Sciences, SPSS (version 15), was used for the following analyses. For measures consisting of two items, bivariate correlations were computed. For those consisting of more than two items, Cronbach's alphas were examined to assess the reliability (internal consistency) of the scales. A principal component analysis (PCA) was also carried out for the designed measures consisting of more than two items to confirm these measures were representing only a single factor or component.

#### Objective 1

All measures were used in path analysis, employing a causal model based on Rogers' Diffusion of Innovations Theory. Simultaneous multiple regression analysis was used to determine the associations between the variables, culminating in the outcome variable of intention to use HHK and with behaviour (frequency of use of HHK) as the model's penultimate outcome. The relative contributions of the mediating variables' (i.e. relative advantage, compatibility, complexity, trialability, and observability) association with intentions and behaviour was also assessed.

#### Objective 2

For those physicians not intending to use HHK (N = 49) contributions of years of experience, Solo vs. Group and Patient encounter duration was separately regressed against individual and environmental barriers.

## Results

Out of the 153 physicians who agreed to participate in the study and received the HHK, 115 survey questionnaires were returned at the two-month follow up (follow-up rate of 75%). Information about the sample is displayed in Table 1.

**Table 1:** Sample characteristics (when not specified n = 115)

Variables		% (n)
Sex		
	Male	52.2 (60)
	Female	47.8 (55)
Year of graduation		
	≤ 1969	11.3 (13)
	1970 – 1989	50.4 (58)
	≥ 1990	38.3 (44)
Practice setting		
	Solo practice	13.9 (16)
	Group practice	71.3 (82)
	Outpatient clinic	14.8 (17)
Academic affiliation		
	Yes	36.5 (42)
	No	63.5 (73)
Time spent in patient care (hours/week)		
	< 20	4.4 (5)
	20 – 40	33.9 (39)
	> 40	61.7 (71)
Average duration of patient encounters (minutes)		
	0 – 10	25.2 (29)
	11 – 20	70.4 (81)
	21 – 30	1.8 (2)
	> 30	2.6 (3)
Personal smoking status (n = 113)		
	Current smoker	1.8 (2)
	Former smoker	17.7 (20)
	Never smoker	80.5 (91)
Personal physical activity level (n = 113)		
	≥ 5 d/wk with 30 min. of mod. intensity PA	35.4 (40)
	< 5 d/wk with 30 min. of mod. intensity PA	64.6 (73)
Purchases low-fat food		
	never/seldom	8.7 (10)
	Occasionally	14.8 (17)
	often/very often	76.5 (88)

The correlations among the study variables are displayed in Table 2. The bivariate correlations of the individual items of Trialability and Observability were .44 (p < .001) and .34 (p < .001) respectively. Cronbach's alpha (α) provided an estimate of strong internal consistency for the measures compatibility (α = .78) and complexity (α = .82). Principal component analysis (PCA) conducted separately for items measuring compatibility and complexity revealed that each of these items had only one eigenvalue greater than one, and scree tests indicated a clear discontinuity between the first and the second components. The components extracted for compatibility and complexity explained 70.2% and 75.3% of the total variance respectively.

**Table 2:** Inter-correlation of variables

	1	2	3	4	5	6	7	8	9	10
1. Behavior										
2. Intention	.66*									
3. Relative advantage	.63 **	.59**								
4. Compatability	.56**	.55**	.70**							
5. Complexity	.56**	.57**	.79**	.84**						
6. Trialability	.44**	.53**	.65**	.60**	.67**					
7. Observability	.55**	.61**	.67**	.66**	.67**	.72				
8. Years of experience	-.16	.01	-.07	-.04	-.03	.04	.01			
9. Solo vs. Group Practices	.15	.13	.06	.07	.19*	.15	.06	-.02		
10. Patient encounter duration	.14	.18	.05	.06	.12	.23*	.20*	.04	.10	

* p < .05; **p < .01

### Objective 1

**Figure 1:**
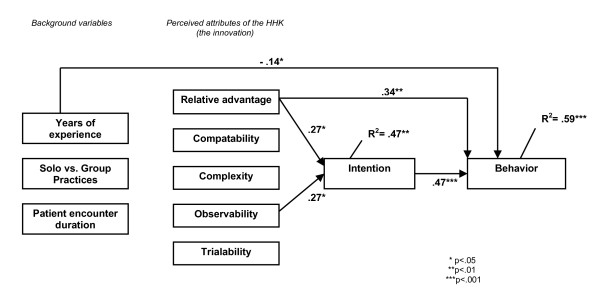
Paths indicating HHK attributes and contextual factors shaping physician intention and behaviour.

Figure 1 presents paths with significant standardized beta coefficients. The coefficients with behaviour as the dependent variable indicate associations with intention (β = 0.47; p < .001), relative advantage (β = 0.34; p < .01), and years of experience (β = -0.14; p < .05), explaining almost 60% of the variance (R^2 ^= .59; p = .001). The variables, relative advantage (β = 0.27; p < .05) and observability (β = 0.27; p < .05) were significantly associated with intentions (R^2 ^= .47; p = .01).

### Objective 2

Solo vs. Group practice was significantly associated with individual barriers (β = - 0.41; p < .05), and environmental barriers (β = - 0.38; p < .05), when controlling for years of experience and patient encounter duration. This indicates that both environmental and individual barriers were higher for individuals practicing as solo physicians.

## Discussion

The decision to adopt an innovation is an active and dynamic process with interactions between the individual, situational factors and contextual factors as well as attributes of the innovation itself. As the work of Denis, Hebert, Langley, Lozeau and Trottier [10] highlights, the people and settings involved in adopting innovations are not rational players, and the advantages or disadvantages of the innovation are distributed unevenly among those involved. That is, adopters bring with them interests, values, and power that further shape and add complexity to the innovation adoption process. Enhancing our understanding of these numerous influencing factors could provide valuable information to guide dissemination efforts and thereby increase the efficiency of innovation implementation. Furthermore, existing social-cognitive theories can help guide investigations in this field of study, especially considering that, in general, theory-based interventions are more efficacious than atheoretical approaches for behaviour change [11].

This study examined the HHK, a kit that was developed to ensure physicians in primary care settings have the latest knowledge about CVD risk factors. Adoption of this kit by physicians practicing in this setting is a first step in decreasing the gap between what is known (research) and what is implemented in clinical practice (practice). The primary care setting is an appropriate point for screening, detecting, monitoring and treating CVD risk factors, and physicians can serve as an immediate portal for CVD health promotion and disease prevention information since they have contact with at least 70% of all adults each year [12]. Research supports the need for a kit such as HHK, as one study demonstrated that only about half of the physicians routinely advise people to quit smoking and only a third of patients who should have treatment for high blood cholesterol receive it [13]. Further, in a large study of 603 CVD patients, 199 patients (33%) with CVD were not screened with lipid panels, 271 patients (45%) were not receiving dietary counselling, and 404 (67%) were not receiving cholesterol medication in accordance with the National Cholesterol Education Program guidelines [14].

Informed by theoretical frameworks [namely, Rogers' Diffusion of Innovation theory, and the intention-behaviour association (based on the TPB)], this research study investigated whether distinct attributes of the HHK as well as contextual and situational factors are associated with physicians' intention and actual usage of the kit, and whether any contextual and situational factors influence individual or environmental barriers that prevent the uptake of the HHK kit. This study found two of its attributes to be more influential than the others, namely relative advantage and observability. Relative advantage is the degree to which an innovation is perceived as being better than the idea it supersedes. The advantage may be conceptualized in terms of economic profitability, social prestige or ease of use. Innovations that have a clear unambiguous advantage over the standard will be more easily adopted and implemented. This study finding is in line with current research evidence from the health sector suggesting that relative advantage is *sine qua non *for innovation adoption, that is, if a potential user sees no advantage in using the innovation it will not be adopted [6]. This finding may be of significance to knowledge translation researchers and designers of health promotion resources and the finding emphasises the importance of having a clear understanding of existing resources when designing new information resources. In other words, new health information innovations must clearly have an 'edge' or advantage over existing resources. It is not surprising that physicians found the HHK to have a relative advantage especially considering that McClaran and colleagues described the HHK to be the most comprehensive tools for CVD health promotion [13], and the Achieving Cardiovascular Health in Canada (ACHIC) partnership endorsed the HHK.

In this study, observability of the benefits of the HHK was another attribute found to be associated with physicians' intention to use it. Although this result is somewhat expected in the area of evidence-based practice, this finding may not be generalizable to other areas of health care practice. Dopson, FitzGerald, Ferlie, Gabbay and Locock [15] state in their meta-synthesis work that there is still a weak relationship between the strength of the evidence base and clinical behaviour change, and there was no discernible pattern that innovations, supported by stronger evidence were diffusing faster. Fitzgerald, Ferlie, Wood and Hawkins [16] also echo this claim when reporting on their findings of two comparative studies exploring eight different innovations in the acute and primary care sectors of healthcare in the United Kingdom.

Our study found that both environmental and individual barriers that prevent the uptake of the HHK among those physicians who do not intend to use the kit were higher for individuals practicing as solo vs those practicing in a group setting indicating that the context within which adoption decisions are made could have an influence in the adoption process. This outcome is in line with a study where physicians, nurses and managers were asked to rate the frequency of their use of a variety of information sources, the frequency of their research use and the factors thought to influence their research use [17], and found the importance of creating opportunities for interaction to enhance research use. Transposed onto our study, these findings suggest that innovation use is enhanced through interaction of potential users. Group medical practices (compared to solo practices) certainly facilitate interaction amongst potential users, thus work context can help shape the innovation adoption process. It then makes intuitive sense that physicians working in solo practices would report more barriers to using or intending to use the HHK. Physicians working in solo practices do not have easy access to colleagues to discuss new information innovations – thus may hinder the ability of dialogue and the social construction of the utility of the HHK. Furthermore, in group medical practices, physicians can share resources and expertise thus freeing up more time to try new innovations, such as the HHK. Another related explanation for why physicians in group practice (who did not intend to use the HHK) reported fewer barriers to innovation use is the proximity to intermediaries. Intermediaries such as opinion leaders, change agents, and knowledge brokers are considered to play an important role in convincing others to adopt an innovation or use research in their practice [18,19]. These intermediaries can be fellow colleagues, thus physicians in group medical practices have an obvious advantage compared to physicians working in solo practices. Thus, the local environment in which a clinician practices is a mediator in the innovation adoption process.

An interesting finding of this study is that years of experience of the physicians were found to be negatively associated with the frequency of use of the HHK kit. This finding perhaps suggests that older physicians are less open to adopting new ideas. Other studies have also shown evidence of this. One study [20] that systematically reviewed data from several studies linked the physician's age or years since graduation with inferior knowledge of the latest cancer-screening techniques, and poorer diagnosis and treatment of other chronic diseases. Out of the studies listed in this review, the most striking is a study that analyzed mortality for 39 007 hospitalized patients with acute myocardial infarction [21] where researchers observed a 0.5% increase in mortality for every year since the treating physician had graduated from medical school.

Strengths of our research include the sizable follow up rate (75%) we had for this study. Considering that family physicians are often a more challenging group of practitioners to reach as they tend not to work in large, academic medical centre, this follow up rate is notable. Conversely, a limitation of this study is that only the short-term adoption of the kit (i.e. two months after dissemination) was examined, thus we do not know if these adoption behaviours continue at the same rate or if there was erosion of the HHK usage rates. On the other hand, perhaps an alternative explanation that requires consideration is that a two month follow up may not have been long enough to fully assess the adoption of the kit. Given the busy clinic schedules of family physicians, perhaps more 'time with' the HHK is needed prior to using it in practice. At this point in time there is no clear indication in the literature with respect to the length of time individuals need to spend with an innovation prior to making the decision to use it. Future studies should investigate if these results hold true for long-term maintenance of behaviour, as factors influencing long-term maintenance of adoption behaviour may be different. Future studies are also encouraged to examine other theoretical constructs that were not employed in this study, and further examine the detailed nature of the concepts (i.e., if physicians conceptualized relative advantage in terms of economic profitability, social prestige or ease of use). The exact natures of the individual and social barriers experienced by physicians for the adoption of new innovations are other areas of study that could be investigated.

## Conclusion

Findings of this study suggest that the innovation adoption process is not straightforward, but attributes of the innovation, contextual factors, and situational factors play an important role in the process. This study, along with work of Fitzgerald and colleagues [16] and Dopson and colleagues [15] suggest that the context in which the adoption occurs shapes and moulds the adoption process. The results of this study specifically suggest that future information innovations, such as patient education kits and practitioner resources need to demonstrate an advantage over current resources and the research evidence needs to be clearly visible. Further, it seems to indicate that the innovation adoption process has a social element, and collegial interactions and discussions may facilitate that process. These results could be valuable for knowledge translation researchers and innovation developers in innovation adoption planning. Future research is encouraged to investigate the nature of this process, as well as examine other theoretical constructs that were not explored in this study.

## Competing interests

The authors declare that they have no competing interests.

## Authors' contributions

SS participated in study conception, data analysis and interpretation and drafted the manuscript. RP conceived the overall project and its design, secured funding, and provided leadership and final approval of the submitted manuscript. NK participated in data analysis and interpretation. RB conceived the study protocol and participated in data collection. WR participated in study conception. All authors read and approved the final manuscript.
